# Dietary index for gut microbiota, a novel protective factor for the prevalence of chronic kidney diseases in the adults: insight from NHANES 2007–2018

**DOI:** 10.3389/fnut.2025.1561235

**Published:** 2025-03-19

**Authors:** Yunfei Xiao, Yaqing Yang, Shunyu Gao, Hao Zhang, Jia Wang, Tao Lin, Yunjin Bai

**Affiliations:** ^1^Department of Urology, Institute of Urology, West China Hospital, Sichuan University, Chengdu, China; ^2^Organ Transplantation Center, West China Hospital, Sichuan University, Chengdu, Sichuan, China; ^3^Department of Respiratory and Critical Care Medicine, West China Hospital, Sichuan University, Chengdu, China

**Keywords:** chronic kidney diseases, dietary index for gut microbiota, metabolism, cross-section, association

## Abstract

**Introduction:**

This study explore the association between the dietary index for gut microbiota (DI-GM) and the prevalence of chronic kidney disease (CKD).

**Method:**

A cross-sectional study of participants aged ≥20 years using the data drawn from NHANES (2007–2018). DI-GM is comprised 14 dietary components (10 beneficial and 4 unfavorable). CKD diagnosis based on urine albumin-to-creatinine ratio (uACR) and estimated glomerular filtration rate (eGFR). Logistic regression models were employed to evaluate the relationship between DI-GM and CKD while controlling for various covariates. Additionally, a spline smooth analysis was performed. Subgroup and interaction analyses were conducted to investigate whether any factors modified this relationship.

**Results:**

A total of 28,843 participants were eligible for the study, of whom 5,461 were diagnosed with CKD, while 23,382 were not. Patients with CKD exhibited significantly lower DI-GM scores compared to healthy individuals. A negative association between DI-GM and the prevalence of CKD was observed across all models, with the relationship being more pronounced in individuals with DI-GM scores greater than 5 compared to those with scores ≤3. Beneficial components, such as dietary fiber, whole grains, and coffee, were identified as protective factors. Moreover, sex make an effect on this relationship, with stronger effects noted in women.

**Conclusion:**

Higher DI-GM scores correlate with reduced CKD prevalence, and the effect appears to be more pronounced in women than in men. These findings suggest that enhancing gut health through diet may serve as a viable strategy for the prevention and management of CKD, with particular attention to sex-based differences in prevention.

## 1 Introduction

Chronic kidney disease (CKD) is a long-term condition characterized by progressive renal dysfunction, leading to impaired filtration and waste elimination. CKD frequently progresses to complications such as cardiovascular disease, anemia, and bone disorders, and may ultimately necessitate dialysis or transplantation. Globally, CKD affects over 850 million people and is projected to become the fifth leading cause of death by 2040 (1). The primary risk factors for CKD include diabetes, hypertension, obesity, and aging (2). Recent studies have highlighted the role of gut dysbiosis in CKD, as it exacerbates systemic inflammation, promotes insulin resistance, and generates uremic toxins like indoxyl sulfate and p-cresyl sulfate (3, 4). These toxins contribute to further kidney damage and cardiovascular complications, highlighting the connection between gut health and CKD progression (5, 6). Consequently, the gut microbiome is increasingly recognized as a potential therapeutic target in the management of CKD.

The dietary index for gut microbiota (DI-GM) is a novel metric designed to assess the impact of diet on the diversity of gut microbiota. This comprehensive index was developed through a review of 106 articles that investigated the association between diet and gut microbiota in adults. The DI-GM encompasses 14 dietary components, with beneficial components such as fermented dairy, whole grains, fiber, and specific fruits and vegetables, and unfavorable components like red and processed meats, refined grains, and high-fat diets (7, 8). This index serves as a standardized tool for evaluating dietary patterns that promote or hinder gut microbiota health. Research indicates that diet affecting gut microbiota diversity can influence systemic health, particularly concerning metabolic conditions such as diabetes and cardiovascular diseases (9, 10). Given the established roles of metabolic disturbances and gut dysbiosis in kidney disease, understanding whether DI-GM correlates with CKD prevalence is essential for developing dietary interventions aimed at preserving renal function. Despite the growing body of evidence linking diet, gut microbiota, and chronic diseases, there is a notable scarcity of research exploring the relationship between DI-GM and CKD. This study aims to investigate the association between the DI-GM and the prevalence of CKD, hypothesizing that higher DI-GM scores, indicative of a gut-friendly diet, are associated with a lower prevalence of CKD. The urgency of this research is emphasized by the global rise in CKD incidence, which is significantly influenced by lifestyle factors, including poor dietary habits and metabolic health. By examining this relationship within a cross-sectional adult population, the study aims to provide insights into how dietary strategies targeting gut microbiota may help mitigate the risk and progression of CKD.

## 2 Method

### 2.1 Study design and population

The data utilized in this research was obtained from the National Health and Nutrition Examination Survey (NHANES) database, which covers the period from 2007 to 2018. NHANES is a comprehensive, nationally representative survey designed to investigate the dietary patterns and health of individuals residing in the United States. Data were collected through structured interviews, physical evaluations, and laboratory tests, employing a multistage probability sampling technique. The primary objective is to accurately represent the demographics of the U.S. population, with stringent measures implemented during the data collection process to ensure the confidentiality and privacy of participants. The initial sample, collected over six consecutive cycles, comprised 59,842 individuals. First, individuals under 20 years of age were excluded from the sample (*n* = 34,770). Next, pregnant individuals were also removed (*n* = 372). Additionally, individuals with missing information regarding CKD diagnoses (*n* = 3,468) and DI-GM (*n* = 2,087) were excluded. Ultimately, 28,843 adults were included in the analysis, as detailed in Supplementary Figure S1.

All NHANES study protocols received approval from the Ethics Review Committee of the NCHS, and informed consent was obtained from all participants.

### 2.2 The dietary index for gut microbiota

The DI-GM is a literature-based index developed to assess dietary patterns that influence gut microbiota composition (7). It encompasses a set of dietary components that either positively or negatively impact gut microbial diversity. This index was constructed using dietary recall data and consists of 14 food components, categorized into beneficial and unfavorable groups based on their effects on gut microbiota diversity (Supplementary Table S1) (8). Beneficial components include foods such as fermented dairy, whole grains, fiber, soybean, broccoli, avocados, cranberries, chickpeas, coffee, and green tea, all of which have been shown to promote a healthier gut microbiome. Conversely, red meat, processed meats, refined grains, and high-fat diets (defined as ≥40% of total energy from fat) are classified as unfavorable due to their association with dysbiosis. For beneficial food components, a score of 1 is assigned when intake meets or exceeds the sex-specific median, indicating sufficient consumption. If intake falls below this threshold, a score of 0 is assigned. For unfavorable components, the scoring is reversed: a score of 1 is assigned if intake is below the median or under 40% of daily energy for high-fat diets, while a score of 0 is given. The total DI-GM score ranges from 0 to 14, with higher scores indicating diets that are more supportive of gut microbiota health. Participants are categorized into four subgroups based on the total score: (1) 0–3 points, (2) 4 points, (3) 5 points, and (4) ≥6 points.

### 2.3 Diagnosis of chronic kidney diseases

In this study, CKD was diagnosed based on the patient’s urine albumin-to-creatinine ratio (uACR) and estimated glomerular filtration rate (eGFR) (11). The uACR was categorized into three levels: A1 (<30 mg/g), A2 (30–300 mg/g), and A3 (>300 mg/g), while the eGFR was classified into six levels: G1 (≥90 mL/min/1.73 m^2^), G2 (60–89 mL/min/1.73 m^2^), G3a (45–59 mL/min/1.73 m^2^), G3b (30–44 mL/min/1.73 m^2^), G4 (15–29 mL/min/1.73 m^2^), and G5 (<15 mL/min/1.73 m^2^). Based on the combination of uACR and eGFR, CKD prognosis was stratified into four risk categories: (1) Low Risk: uACR <30 mg/g (A1) with eGFR ≥60 mL/min/1.73 m^2^ (G1 or G2), indicating minimal risk of kidney damage; (2) Moderate Risk: uACR <30 mg/g (A1) with eGFR 45–59 mL/min/1.73 m^2^ (G3a), or uACR 30–300 mg/g (A2) with eGFR ≥60 mL/min/1.73 m^2^, suggesting a moderate risk of kidney damage; (3) High Risk: uACR <30 mg/g (A1) with eGFR 30–44 mL/min/1.73 m^2^ (G3b), or uACR 30–300 mg/g (A2) with eGFR 45–59 mL/min/1.73 m^2^, or uACR >300 mg/g (A3) with eGFR ≥60 mL/min/1.73 m^2^, indicating significant kidney damage; (4) Very High Risk: eGFR <30 mL/min/1.73 m^2^, or uACR >30 mg/g with eGFR 30–59 mL/min/1.73 m^2^, indicating severe kidney damage and a poor prognosis. This stratification facilitates a precise assessment of CKD prognosis, confirming a diagnosis of CKD (‘yes’) for moderate or higher risk categories.

### 2.4 Covariates

The continuous variables included age, poverty income ratio (PIR, a ratio of family income to poverty threshold), and body mass index (BMI). The categorical variables encompassed sex, race, education, marital status, smoking status (never, former, or current), alcohol consumption (never, former, or current), diabetes, cardiovascular diseases (CVD), hypertension (HBP), cancer, vigorous activity, and moderate activity (all classified as yes/no). Specifically, age was categorized into the intervals of 20–34, 35–49, 50–64, and ≥ 65 years, while racial categories included Mexican American, other Hispanic, Non-Hispanic Black, non-Hispanic White, and other races. Participants were classified based on BMI into the following groups: <25, ≥25 & <30, and ≥ 30 kg/m^2^.

### 2.5 Statistical analysis

The baseline characteristics of all participants were presented using means ± standard deviations (SD) and proportions. Specifically, continuous variables were evaluated using a linear regression model, while categorical variables were analyzed using chi-square tests. To identify independent risk factors associated with CKD, logistic regression analyses were conducted. Participants were categorized into four groups based on the DI-GM, and three logistic regression models were developed to investigate the relationship between DI-GM and CKD. Furthermore, to assess the association between the components of DI-GM and the prevalence of CKD among adults, three additional logistic regression models were employed. In the unadjusted model, no variables were modified. The minimally adjusted model controlled for age, sex, race, PIR, BMI, marital status, and education. The fully adjusted model further included smoking habits, alcohol consumption, HBP, diabetes, CVD, cancer, vigorous activity, and moderate activity. Additionally, spline smoothing using a generalized additive model (GAM) was performed to illustrate whether a linear relationship between DI-GM and CKD present in the fully adjusted model. To further investigate whether any factors could influence the association between these two variables, interaction and subgroup analyses were conducted.

Moreover, sensitivity analyses of multivariable logistic regression were performed. The DI-GM was based on dietary information collected from a day-two recall. All statistical analyses were conducted using R version 4.2.2 (the R Foundation)^1^ and EmpowerStats (X&Y Solutions, Inc.).^2^ A *p*-value of less than 0.05 was considered statistically significant.

## 3 Results

### 3.1 Baseline characteristics of study participants

**Figure 1:**
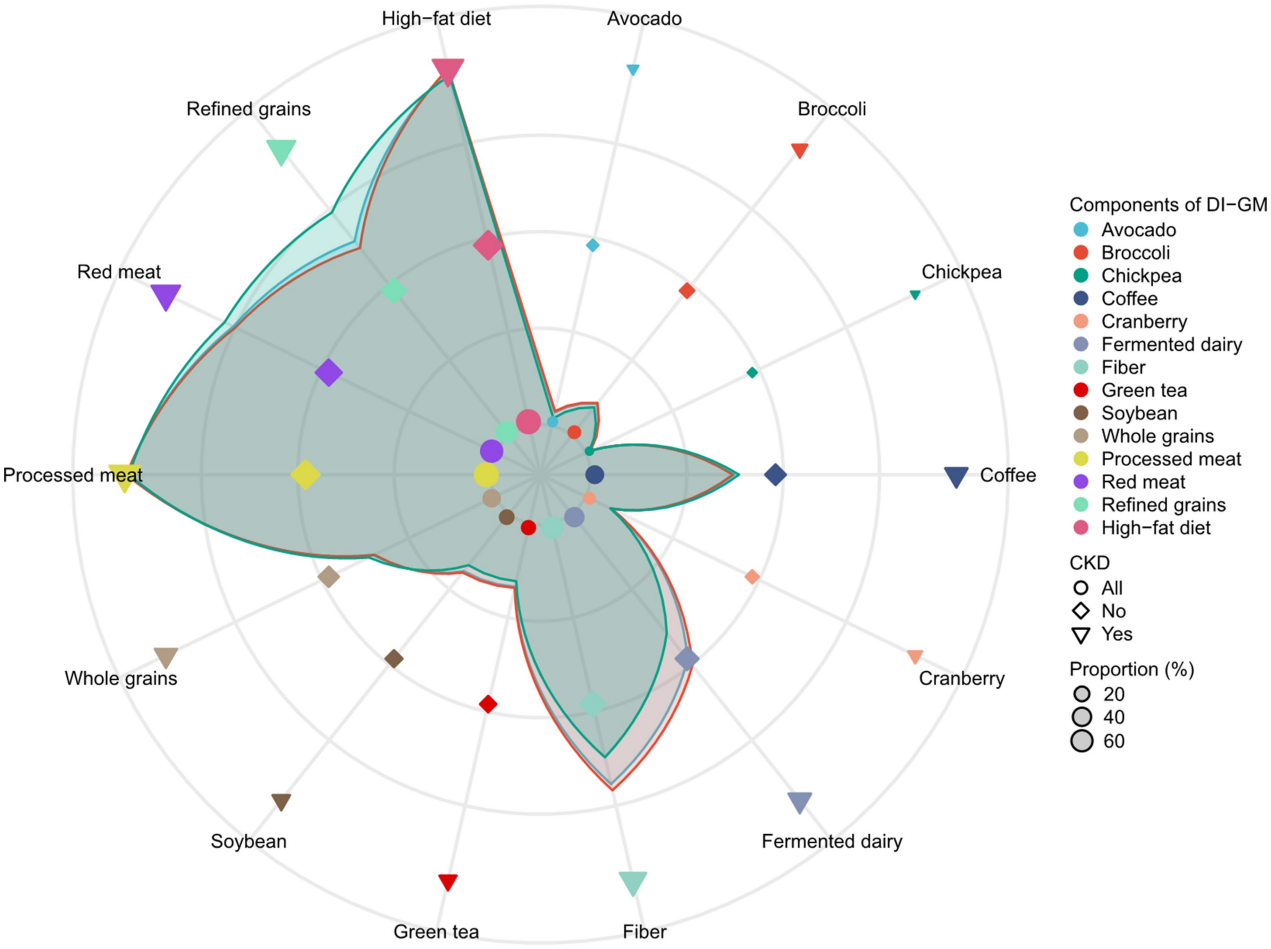
The distributions of dietary index for gut microbiota (DI-GM) components by categories of chronic kidney diseases (CKD).

A total of 28,843 individuals were enrolled in the NHANES study from 2007 to 2018, among which 5,461 participants were diagnosed with CKD while 23,382 were not. As presented in Table 1, individuals with CKD exhibited a higher unfavorable to gut microbiota score (2.71 ± 1.07) and a lower DI-GM (4.54 ± 1.61) and beneficial to gut microbiota score (1.83 ± 1.32) compared to those without CKD. In comparison to healthy participants, those with CKD were older (63.07 ± 16.15 years), with a significant proportion aged ≥65 years (55.01%), and had a higher BMI (30.27 ± 7.33) with 45.50% classified as obese (≥30 kg/m^2^). Additionally, the proportion of females (52.10%), individuals with low education levels (less than college, 44.79%), smokers (49.59%), and those with diabetes (50.25%), HBP (72.77%), CVD (27.63%), and cancer (17.92%) was higher among CKD patients compared to those without. Conversely, participants with CKD had a lower PIR (2.26 ± 1.51 vs. 2.56 ± 1.64) and were less likely to be in non-single living situations (married/living with a partner, 53.98%), engage in moderate physical activity (29.88%), vigorous activity (13.50%), and consume alcohol (81.95%) when compared to healthy individuals. Regarding the components of the DI-GM, we observed that the scores for components beneficial to gut microbiota, such as avocado, broccoli, chickpeas, cranberries, fermented dairy, fiber, green tea, and soybeans, were predominantly lower in CKD patients, while the scores for components unfavorable to gut microbiota, specifically red meat and refined grains, were higher in this group (Supplementary Table S2 and Figure 1).

**Table 1:** Characteristics of participants by categories of Chronic kidney diseases: NHANES 2007–2018.^*^

Variables	All (*n*=28843)	Chronic kidney diseases		*p*-value
		No (*n*=23382)	Yes (*n*=5461)	
DI-GM (mean ± SD)	4.62 ± 1.61	4.64 ± 1.62	4.54 ± 1.61	<0.001
Beneficial to gut microbiota (mean ± SD)	1.99 ± 1.34	2.03 ± 1.34	1.83 ± 1.32	<0.001
Unfavorable to gut microbiota (mean ± SD)	2.63 ± 1.06	2.61 ± 1.06	2.71 ± 1.07	<0.001
Age (years, mean ± SD)	49.97 ± 17.59	46.91 ± 16.47	63.07 ± 16.15	<0.001
20–34 (%)	24.18	28.05	7.62	
35–49 (%)	25.15	28.09	12.58	
50–64 (%)	26.62	27.05	24.79	
≥65 (%)	24.04	16.81	55.01	
PIR (mean ± SD)	2.50 ± 1.62	2.56 ± 1.64	2.26 ± 1.51	<0.001
≤1.3 (%)	32.08	31.33	35.28	
>1.3 and ≤ 3.5 (%)	37.72	36.74	41.95	
>3.5 (%)	30.20	31.93	22.77	
BMI (kg/m^2^, mean ± SD)	29.29 ± 6.94	29.07 ± 6.83	30.27 ± 7.33	<0.001
<25 (%)	28.16	29.20	23.61	
≥25 and < 30 (%)	32.95	33.43	30.89	
≥30 (%)	38.89	37.37	45.50	
Sex (%)				0.026
Female	50.74	50.42	52.10	
Male	49.26	49.58	47.90	
Race (%)				<0.001
Mexican American	15.29	15.86	12.87	
Other Hispanic	42.21	41.11	46.90	
Non-Hispanic White	20.71	20.17	23.00	
Non-Hispanic Black	10.52	10.92	8.79	
Other races	11.27	11.94	8.44	
Education (%)				<0.001
Less than 9th grade	10.30	9.21	14.99	
9–11th grade	13.90	13.43	15.91	
High school graduate	22.89	22.54	24.40	
Some college	29.71	30.22	27.54	
College graduate or above	23.19	24.60	17.16	
Marital (%)				<0.001
Married/Living with partner	59.49	60.77	53.98	
Divorced/Separated/Widowed	22.41	19.42	35.21	
Never married	18.10	19.80	10.81	
Vigorous activity (%)				<0.001
No	79.94	78.41	86.50	
Yes	20.06	21.59	13.50	
Moderate activity (%)				<0.001
No	62.67	60.93	70.12	
Yes	37.33	39.07	29.88	
Alcohol (%)				<0.001
Never	14.15	13.26	18.05	
Former	15.83	13.71	25.15	
Yes	70.02	73.03	56.80	
Smoke (%)				<0.001
Never	55.44	56.62	50.41	
Former	24.32	22.35	32.77	
Yes	20.23	21.03	16.82	
Diabetes (%)				<0.001
No	71.82	76.98	49.75	
Borderline	8.58	8.59	8.55	
Yes	19.60	14.43	41.70	
HBP (%)				<0.001
No	56.63	63.50	27.23	
Yes	43.37	36.50	72.77	
CVD (%)				<0.001
No	88.69	92.50	72.37	
Yes	11.31	7.50	27.63	
Cancer (%)				<0.001
No	90.31	92.23	82.08	
Yes	9.69	7.77	17.92	

* Mean + SD for continuous variables, and *p* value was calculated by weighted t test. % for categorical variables, and *p* value was calculated by weighted chi-square test. SD, Standard Deviation; BMI, Body Mass Index; PIR, Poverty Income Ratio; CVD, Cardiovascular Disease; HBP, Hypertension; DI-GM, dietary index for gut microbiota.

### 3.2 Multivariable logistic regression

Table 2 demonstrates a negative association between DI-GM and the prevalence of CKD across all models. In the non-adjusted model, the odds ratio (OR) was 0.964 (95% CI, 0.946 to 0.982), in the minimally-adjusted model, it was 0.919 (95% CI, 0.901 to 0.937), and in the fully-adjusted model, the OR was 0.958 (95% CI, 0.936 to 0.980). Furthermore, individuals with a DI-GM score > 5 exhibited a significantly higher prevalence of CKD compared to those in the ≤3 score group (OR = 0.837, 95% CI, 0.754 to 0.928). Additionally, the P vale of trend test analyses was <0.5 in all three models.

**Table 2:** Association between DI-GM and the prevalence of CKD in the adults.

Variables (%)	Non-adjusted model^*^		Minimally-adjusted model^**^		Fully-adjusted model^***^	
	OR (95%CI)	*p*	OR (95%CI)	*p*	OR (95%CI)	*p*
DI-GM	0.964 (0.946, 0.982)	<0.001	0.919 (0.901, 0.937)	<0.001	0.958 (0.936, 0.980)	<0.001
Category of DI-GM						
≤3	Ref		Ref		Ref	
>3, and ≤ 4	0.912 (0.839, 0.991)	0.030	0.887 (0.810, 0.971)	0.009	0.938 (0.845, 1.042)	0.235
>4, and ≤ 5	0.924 (0.849, 1.006)	0.070	0.848 (0.773, 0.930)	<0.001	0.939 (0.844, 1.044)	0.245
>5	0.863 (0.795, 0.936)	<0.001	0.699 (0.639, 0.764)	<0.001	0.837 (0.754, 0.928)	<0.001
P for trend	0.001		<0.001		0.001	
Beneficial to gut microbiota	0.894 (0.874, 0.915)	<0.001	0.878 (0.857, 0.900)	<0.001	0.923 (0.897, 0.950)	<0.001
Unfavorable to gut microbiota	1.095 (1.064, 1.126)	<0.001	1.002 (0.973, 1.033)	0.876	1.022 (0.987, 1.059)	0.220

CI, confidence interval; OR, odds ratio; CKD, chronic kidney diseases; DI-GM, dietary index for gut microbiota.

* Non-adjusted model adjusts for none.

** Minimally-adjusted model adjusts for age, sex, race, body mass index, race, poverty income ratio, education, marital.

*** Fully-adjusted model adjusts for age, sex, body mass index, race, poverty income ratio, education, marital, alcohol, smoke, hypertension, cardiovascular disease, cancer, diabetes, moderate activity, vigorous activity.

Furthermore, the results showed that beneficial to gut microbiota serves as a protective factor for CKD (OR = 0.923, 95% CI 0.897 to 0.950), with no significant differences observed in the unfavorable to gut microbiota within the fully adjusted model. Additionally, components of beneficial to gut microbiota, such as coffee, fiber, and whole grains, play a crucial role in protecting CKD across the three models (Table 3).

**Table 3:** Association between the components of DI-GM and the prevalence of CKD in the adults.

HEI-2015 Components	Non-adjusted model^*^		Minimally-adjusted model^**^		Fully-adjusted model^***^	
	OR (95%CI)	*p*	OR (95%CI)	*p*	OR (95%CI)	*p*
Beneficial to gut microbiota						
Avocado	0.505 (0.404, 0.631)	<0.001	0.752 (0.589, 0.961)	0.023	0.866 (0.670, 1.119)	0.272
Broccoli	0.855 (0.766, 0.955)	0.006	0.934 (0.822, 1.061)	0.291	1.001 (0.873, 1.148)	0.989
Chickpea	0.751 (0.552, 1.022)	0.068	0.970 (0.670, 1.404)	0.871	0.923 (0.621, 1.373)	0.694
Coffee	1.063 (0.997, 1.133)	0.063	0.844 (0.782, 0.910)	<0.001	0.843 (0.776, 0.915)	<0.001
Cranberry	0.962 (0.847, 1.091)	0.545	1.060 (0.914, 1.229)	0.441	1.087 (0.924, 1.278)	0.315
Fermented dairy	0.689 (0.647, 0.733)	<0.001	0.960 (0.892, 1.033)	0.271	0.949 (0.877, 1.027)	0.191
Fiber	0.754 (0.711, 0.800)	<0.001	0.806 (0.751, 0.864)	<0.001	0.821 (0.761, 0.885)	<0.001
Green tea	0.883 (0.807, 0.965)	0.006	0.922 (0.833, 1.020)	0.114	0.921 (0.827, 1.027)	0.139
Soybean	0.855 (0.785, 0.931)	<0.001	0.927 (0.842, 1.022)	0.129	0.938 (0.845, 1.041)	0.230
Whole grains	1.064 (0.997, 1.135)	0.063	0.880 (0.815, 0.949)	<0.001	0.865 (0.796, 0.939)	<0.001
Unfavorable to gut microbiota						
Processed meat	0.973 (0.909, 1.042)	0.433	0.966 (0.892, 1.046)	0.392	0.986 (0.905, 1.074)	0.743
Red meat	1.111 (1.045, 1.180)	<0.001	0.990 (0.923, 1.062)	0.778	0.993 (0.921, 1.072)	0.864
Fat	0.928 (0.867, 0.993)	0.031	1.008 (0.932, 1.091)	0.840	1.042 (0.957, 1.135)	0.341
Refined grains	1.462 (1.377, 1.552)	<0.001	1.058 (0.987, 1.135)	0.114	1.062 (0.984, 1.145)	0.121

CI, confidence interval; OR, odds ratio.

* Non-adjusted model adjusts for none.

** Minimally-adjusted model adjusts for age, sex, race, body mass index, race, poverty income ratio, education, marital.

*** Fully-adjusted model adjusts for age, sex, body mass index, race, poverty income ratio, education, marital, alcohol, smoke, hypertension, cardiovascular disease, cancer, diabetes, moderate activity, vigorous activity.

### 3.3 A smooth spline curve

The results demonstrate a negative linear association between DI-GM and the prevalence of CKD, as illustrated in Supplementary Figure S2. Specifically, the dose–response relationship indicates that higher levels of DI-GM are correlated with a lower prevalence of CKD.

### 3.4 Subgroup and interaction analysis

Our findings indicate that sex significantly influenced the relationship under investigation (Supplementary Figure S3). Specifically, the stratified analysis revealed notable differences in effects based on sex, with a *p*-value of 0.043 for the interaction, and the association was more pronounced in female. No other significant interactions were observed among the other variables.

### 3.5 Sensitivity analysis

Based on the dietary information collected on day two for DI-GM, the logistic regression analysis revealed consistent results, demonstrating a negative association between the two (Supplementary Table S3).

## 4 Discussion

This study utilized NHANES data from 2007 to 2018, encompassing a sample of 28,843 adults, to evaluate the association between DI-GM and the prevalence of CKD. Baseline characteristics of the study population indicated that patients with CKD had a higher median age and a greater proportion of individuals with high BMI, HBP, and diabetes. The results demonstrated a linear negative association between DI-GM and the prevalence of CKD, with sex influencing this relationship. Additionally, higher scores of beneficial to gut microbiota were significantly associated with a lower prevalence of CKD. Furthermore, specific dietary components within the beneficial to gut microbiota, such as dietary fiber, coffee, and whole grains, exhibited potential protective effects against CKD.

The above mentioned results underscore the critical role of healthy intestinal flora in preserving kidney health. Recent research suggests that an imbalance in intestinal flora can lead to increased production of uremic toxins, such as indoxyl sulfate and p-cresol sulfate (12). These toxins are detrimental to renal tubular cells and exacerbate CKD progression by promoting systemic inflammation (13, 14). Studies have shown that CKD patients experience a marked reduction in intestinal flora diversity, with this imbalance closely linked to elevated urinary toxin production (15). Moreover, a high DI-GM dietary pattern, which is abundant in dietary fiber and prebiotics, promotes the production of short-chain fatty acids (SCFAs), such as butyrate. These metabolites have been demonstrated to exert anti-inflammatory effects by inhibiting pro-inflammatory cytokines, including TNF-*α* and IL-6, and by mitigating oxidative stress (16, 17). Andrade-Oliveira et al. emphasized that butyrate plays a vital regulatory role in the gut-kidney axis, particularly in cases of ischemia–reperfusion-induced acute kidney injury, suggesting that SCFA production may help delay disease progression in CKD patients (18). This finding indicates that enhancing DI-GM scores, for instance, through increased dietary fiber intake, could offer renal protection for CKD patients. Clinically, improving the DI-GM score may contribute to delaying CKD progression. Future studies should further explore the potential of SCFAs as prognostic biomarkers for CKD and examine the varying protective effects of different types of intestinal flora on CKD.

This study found that sex significantly moderates the relationship between DI-GM and CKD, and a high DI-GM diet offers a more pronounced protective effect against CKD in women, potentially linked to estrogen’s role in regulating intestinal flora and barrier function. Gomez et al. highlighted that estrogen enhances the intestinal barrier by upregulating tight junction proteins, such as occludin, which reduces the production of pro-inflammatory uremic toxins and mitigates kidney damage (19, 20). Furthermore, women typically exhibit greater diversity in gut microbiota, which may enhance the benefits derived from a high DI-GM diet (21, 22). In contrast, men often favor high-fat and high-protein diets, which can disrupt bacterial balance and elevate pro-inflammatory bacteria, thereby diminishing the protective effects of DI-GM (23, 24). This study underscores the importance of considering sex differences in the dietary management of CKD patients, particularly for women who may experience greater advantages from a high DI-GM diet. Future personalized strategies should be developed to address these sex-specific dietary needs. Additionally, further research is warranted to elucidate the mechanisms by which estrogen contributes to intestinal barrier integrity and renal protection, thereby providing a more precise foundation for nutritional interventions targeting female CKD patients.

This study also analyzed the role of specific food components in dietary interventions for CKD and found that dietary fiber, coffee, and whole grains significantly contribute to kidney protection. As a prebiotic, dietary fiber promotes the proliferation of beneficial bacteria, such as Bifidobacteria, produces SCFAs, reduces pro-inflammatory factors, and enhances the intestinal environment (25). Research highlighted that dietary fiber intake is significantly associated with slowing the progression of CKD, with its mechanism closely linked to the improvement of bacterial flora diversity and the anti-inflammatory effects of dietary fiber (26). Furthermore, whole grain consumption has been shown to lower levels of chronic inflammatory markers, such as C-reactive protein, while improving metabolic health (27). Antioxidant components, such as polyphenols found in coffee, are also recognized for their protective effects against CKD. Research indicated that moderate coffee intake is associated with a deceleration in the progression of CKD, further corroborating the anti-inflammatory effects of coffee (28–30). This study provides specific guidance for the dietary management of CKD patients, suggesting that increasing the intake of dietary fiber, whole grains, and moderate amounts of coffee may enhance kidney protection. Future research should further explore the specific protective effects of these dietary components on CKD to optimize dietary strategies for renal health.

This study presents both strengths and weaknesses. It utilizes the NHANES large-scale database, which boasts a substantial sample size and broad representativeness, thereby enhancing the feasibility and generalizability of the results. Additionally, the study is noteworthy for being the first to analyze the correlation between DI-GM and CKD, providing a novel reference point for dietary interventions in CKD patients. However, the cross-sectional design of this study presents challenges in establishing a causal relationship between the two variables. Furthermore, the NHANES database lacks specific data on intestinal flora, which limits further investigation into the role of intestinal microorganisms in the mechanisms underlying CKD. Future research should incorporate longitudinal studies alongside microbiome technologies to elucidate the long-term effects of DI-GM on intestinal flora regulation and the protection of renal function.

## 5 Conclusion

This study provides compelling evidence that higher DI-GM scores are associated with a reduced prevalence of CKD in adults, with the protective effect being more pronounced in women than in men. Our findings highlight the significant contributions of beneficial dietary components, such as dietary fiber, whole grains, and coffee, which are integral to a gut-healthy diet. These results suggest that enhancing gut health through diet may serve as a viable strategy for the prevention and management of CKD, particularly by focusing on sex-based differences in prevention efforts. Future prospective cohort studies and microbiome analyses are warranted to further elucidate the role of gut microbiota in the progression of CKD and to support the development of more precise, individualized dietary intervention strategies.

## Data Availability

The datasets presented in this study can be found in online repositories. The names of the repository/repositories and accession number(s) can be found at: https://www.cdc.gov/nchs/nhanes/index.htm.

## Footnotes

^1^ http://www.R-project.org

^2^ http://www.empowerstats.com

## Supplementary material

SUPPLEMENTARY FIGURE S1 Flow diagram of obtaining the final inclusion in the population.

SUPPLEMENTARY FIGURE S2 A spline smoothing demonstrated the linear association between DI-GM and the prevalence of CKD.

SUPPLEMENTARY FIGURE S3 Logistic regression analysis to identify variables that modify the correlation between DI-GM and the prevalence of CKD.
