# Supplementary figures and images for: Dietary index for gut microbiota, a novel protective factor for the prevalence of chronic kidney diseases in the adults: insight from NHANES 2007–2018
Source: Front Nutr. 2025 Mar 19;12:1561235. doi: 10.3389/fnut.2025.1561235 (PMC11963806; doi:10.3389/fnut.2025.1561235)

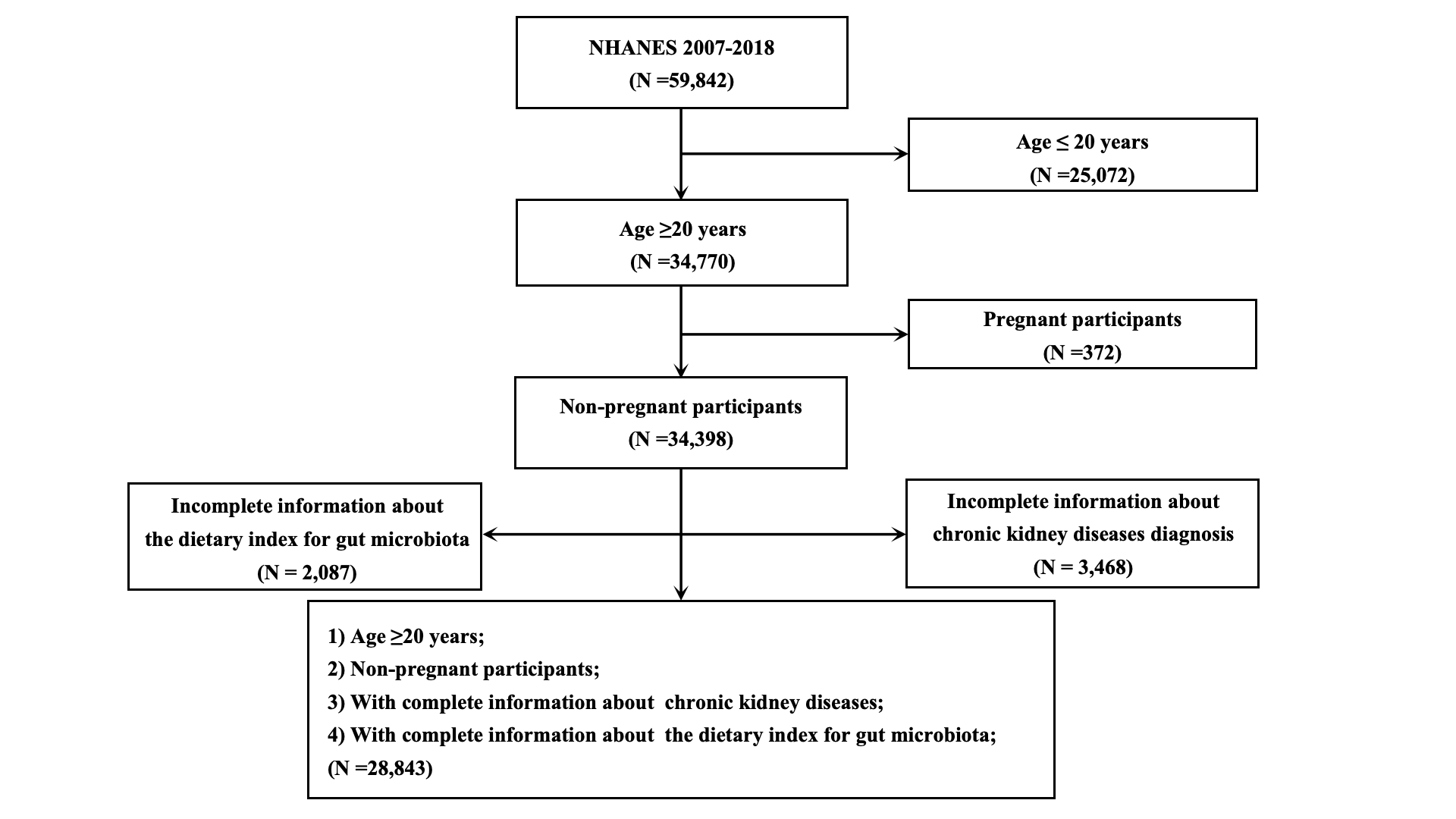

Supplement: SUPPLEMENTARY FIGURE S1 — Flow diagram of obtaining the final inclusion in the population. [file Image_1.tif]

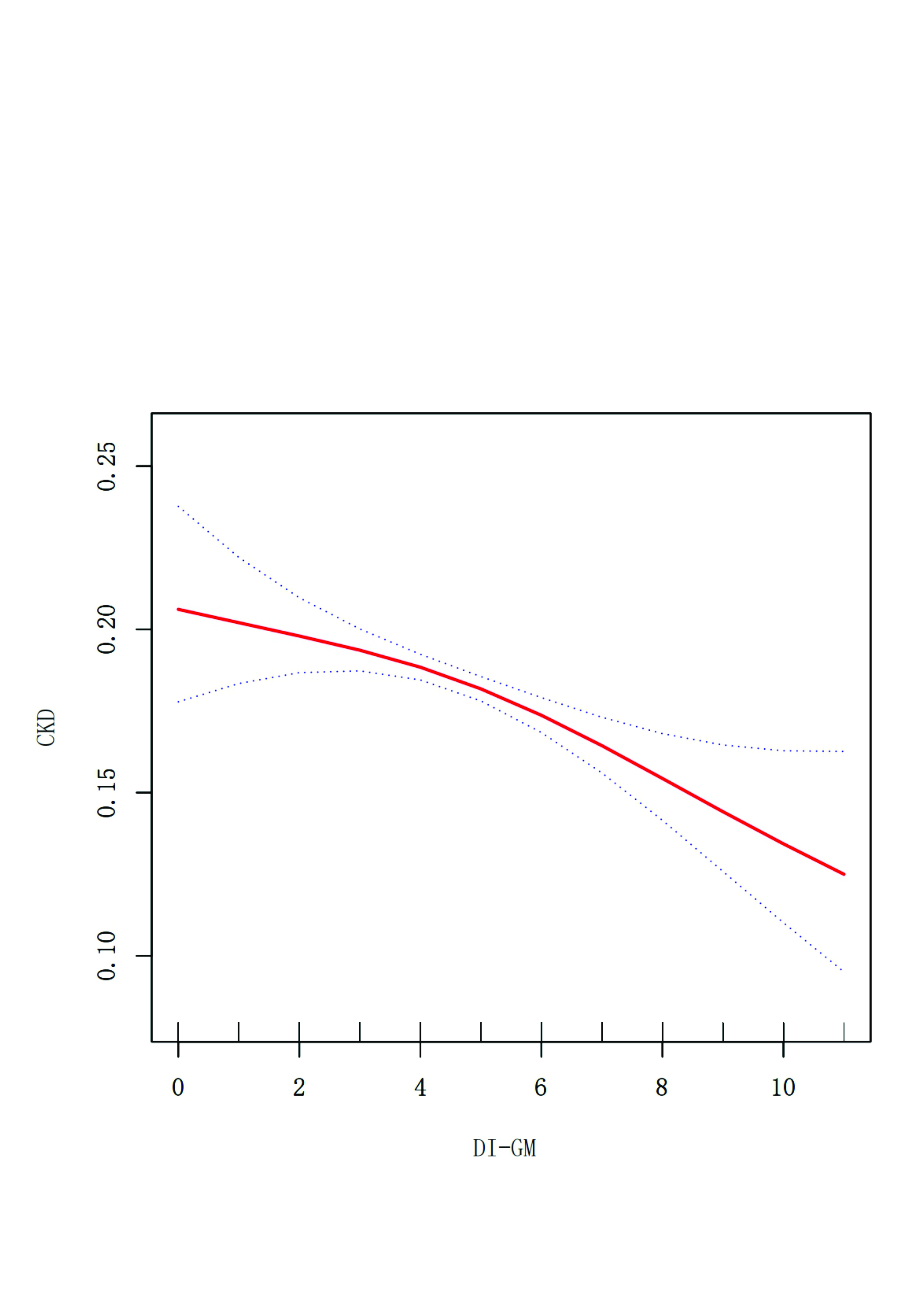

Supplement: SUPPLEMENTARY FIGURE S2 — A spline smoothing demonstrated the linear association between DI-GM and the prevalence of CKD. [file Image_2.tiff]

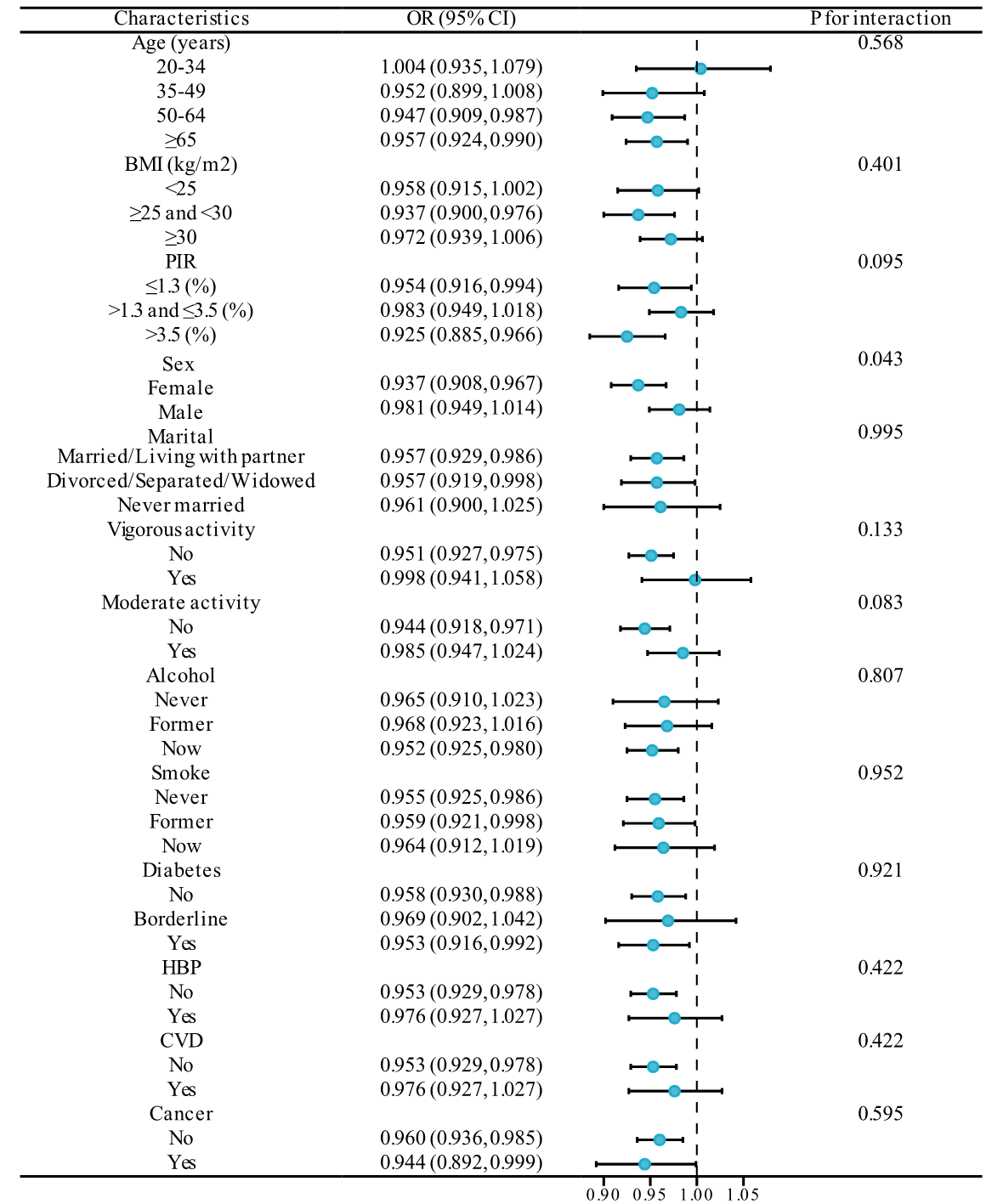

Supplement: SUPPLEMENTARY FIGURE S3 — Logistic regression analysis to identify variables that modify the correlation between DI-GM and the prevalence of CKD. [file Image_3.tif]
