# Supplementary material for: Dietary index for gut microbiota, a novel protective factor for the prevalence of chronic kidney diseases in the adults: insight from NHANES 2007–2018
Source: Front Nutr. 2025 Mar 19;12:1561235. doi: 10.3389/fnut.2025.1561235 (PMC11963806; doi:10.3389/fnut.2025.1561235)
Supplement: Supplementary file 4 [file Table_1.docx]

**Table S1. Components and diagnosis details of the DI-GM.**

| **Components of DI-GM** | **Scoring details** |
| --- | --- |
| **Beneficial to gut microbiota** |  |
| Avocado | For each components:  Score 1. Consumption ≥ the sex-specific median,  Score 0. Otherwise. |
| Broccoli |  |
| Chickpea |  |
| Coffee |  |
| Cranberry |  |
| Fermented dairy |  |
| Fiber |  |
| Green tea |  |
| Soybean |  |
| Whole grains |  |
| **Unfavorable to gut microbiota** |  |
| Processed meat | For each components:  Score 0. Consumption ≥ the sex-specific median,  Score 1. Otherwise. |
| Red meat |  |
| Refined grains |  |
| High-fat diet | Score 0. Consumption ≥ 40%,  Score 1. Otherwise. |
