# Supplementary material for: Dietary index for gut microbiota, a novel protective factor for the prevalence of chronic kidney diseases in the adults: insight from NHANES 2007–2018
Source: Front Nutr. 2025 Mar 19;12:1561235. doi: 10.3389/fnut.2025.1561235 (PMC11963806; doi:10.3389/fnut.2025.1561235)
Supplement: Supplementary file 5 [file Table_2.docx]

**Table S2. The components of DI-GM in the participants categoried by Chronic kidney diseases.***

| **Variables** | **All**  **(n=28843)** | **Chronic kidney diseases** | | **P** |
| --- | --- | --- | --- | --- |
|  |  | **No**  **(n=23382)** | **Yes**  **(n=5461)** |  |
| **DI-GM (mean ± SD,** **%)** | 4.62 ± 1.61 | 4.64 ± 1.62 | 4.54 ± 1.61 | <0.001 |
| ≤3 | 24.06 | 23.68 | 25.69 |  |
| >3, and ≤4 | 24.79 | 24.84 | 24.57 |  |
| >4, and ≤5 | 23.02 | 23.00 | 23.07 |  |
| >5 | 28.13 | 28.47 | 26.66 |  |
| **Beneficial to gut microbiota**  **( mean ± SD)** | 1.99 ± 1.34 | 2.03 ± 1.34 | 1.83 ± 1.32 | <0.001 |
| **Unfavorable to gut microbiota**  **(mean ± SD)** | 2.63 ± 1.06 | 2.61 ± 1.06 | 2.71 ± 1.07 | <0.001 |
| **Beneficial to gut microbiota** |  |  |  |  |
| Avocado (%) |  |  |  | <0.001 |
| Score 0 | 97.15 | 96.86 | 98.39 |  |
| Score 1 | 2.85 | 3.14 | 1.61 |  |
| Broccoli (%) |  |  |  | 0.006 |
| Score 0 | 91.57 | 91.35 | 92.51 |  |
| Score 1 | 8.43 | 8.65 | 7.49 |  |
| Chickpea (%) |  |  |  | 0.067 |
| Score 0 | 98.89 | 98.83 | 99.12 |  |
| Score 1 | 1.11 | 1.17 | 0.88 |  |
| Coffee (%) |  |  |  | 0.063 |
| Score 0 | 70.22 | 70.46 | 69.18 |  |
| Score 1 | 29.78 | 29.54 | 30.82 |  |
| Cranberry (%) |  |  |  | 0.544 |
| Score 0 | 94.11 | 94.07 | 94.29 |  |
| Score 1 | 5.89 | 5.93 | 5.71 |  |
| Fermented dairy (%) |  |  |  | <0.001 |
| Score 0 | 61.43 | 59.81 | 68.36 |  |
| Score 1 | 38.57 | 40.19 | 31.64 |  |
| Fiber (%) |  |  |  | <0.001 |
| Score 0 | 44.60 | 43.28 | 50.28 |  |
| Score 1 | 55.40 | 56.72 | 49.72 |  |
| Green tea (%) |  |  |  | 0.006 |
| Score 0 | 86.57 | 86.30 | 87.71 |  |
| Score 1 | 13.43 | 13.70 | 12.29 |  |
| Soybean (%) |  |  |  | <0.001 |
| Score 0 | 84.80 | 84.43 | 86.38 |  |
| Score 1 | 15.20 | 15.57 | 13.62 |  |
| Whole grains (%) |  |  |  | 0.063 |
| Score 0 | 71.85 | 72.09 | 70.83 |  |
| Score 1 | 28.15 | 27.91 | 29.17 |  |
| **Unfavorable to gut microbiota** |  |  |  |  |
| Processed meat (%) |  |  |  | 0.433 |
| Score 0 | 24.53 | 24.43 | 24.94 |  |
| Score 1 | 75.47 | 75.57 | 75.06 |  |
| Red meat (%) |  |  |  | <0.001 |
| Score 0 | 39.62 | 40.09 | 37.59 |  |
| Score 1 | 60.38 | 59.91 | 62.41 |  |
| High-fat diet (%) |  |  |  | 0.031 |
| Score 0 | 24.34 | 24.08 | 25.47 |  |
| Score 1 | 75.66 | 75.92 | 74.53 |  |
| Refined grains (%) |  |  |  | <0.001 |
| Score 0 | 48.50 | 50.28 | 40.89 |  |
| Score 1 | 51.50 | 49.72 | 59.11 |  |
