# Supplementary material for: Dietary index for gut microbiota, a novel protective factor for the prevalence of chronic kidney diseases in the adults: insight from NHANES 2007–2018
Source: Front Nutr. 2025 Mar 19;12:1561235. doi: 10.3389/fnut.2025.1561235 (PMC11963806; doi:10.3389/fnut.2025.1561235)
Supplement: Supplementary file 6 [file Table_3.docx]

**Table S3 Association between DI-GM (day two recall) and the prevalence of CKD in the adults.**

| **Variables (%)** | **Non-adjusted model*** | | **Minimally-adjusted model**** | | **Fully-adjusted model***** | |
| --- | --- | --- | --- | --- | --- | --- |
|  | **OR (95%CI)** | **P** | **OR (95%CI)** | **P** | **OR (95%CI)** | **P** |
| **DI-GM** | 0.956 (0.937, 0.974) | <0.001 | 0.936 (0.915, 0.958) | <0.001 | 0.944 (0.921, 0.967) | <0.001 |
| **Category of DI-GM** |  |  |  |  |  |  |
| ≤3 | Ref |  | Ref |  | Ref |  |
| >3, and ≤4 | 0.955 (0.874, 1.043) | 0.303 | 0.947 (0.854, 1.050) | 0.300 | 0.974 (0.872, 1.089) | 0.645 |
| >4, and ≤5 | 0.936 (0.855, 1.024) | 0.149 | 0.900 (0.810, 1.000) | 0.049 | 0.932 (0.832, 1.044) | 0.224 |
| >5 | 0.849 (0.779, 0.927) | <0.001 | 0.765 (0.691, 0.848) | <0.001 | 0.801 (0.717, 0.894) | <0.001 |
| **P for trend** | <0.001 | | <0.001 | | 0.001 | |
| **Beneficial to gut microbiota** | 0.884 (0.862, 0.906) | <0.001 | 0.897 (0.871, 0.924) | <0.001 | 0.903 (0.875, 0.932) | <0.001 |
| **Unfavorable to gut microbiota** | 1.078 (1.046, 1.111) | <0.001 | 0.999 (0.965, 1.034) | 0.932 | 1.009 (0.972, 1.047) | 0.630 |

CI: confidence interval, OR: odds ratio, CKD: chronic kidney diseases, DI-GM: dietary index for gut microbiota.

*Non-adjusted model adjusts for none. 
** Minimally-adjusted model adjusts for age, sex, race, body mass index, race, poverty income ratio, education, marital,
*** Fully-adjusted model adjusts for age, sex, body mass index, race, poverty income ratio, education, marital, alcohol, smoke, hypertension, cardiovascular disease, cancer, diabetes, moderate activity, vigorous activity.
